# Extracellular DNA: A Nutritional Trigger of *Mycoplasma bovis* Cytotoxicity

**DOI:** 10.3389/fmicb.2019.02753

**Published:** 2019-11-29

**Authors:** Xifang Zhu, Emilie Dordet-Frisoni, Lucie Gillard, Abou Ba, Marie-Claude Hygonenq, Eveline Sagné, Laurent Xavier Nouvel, Renaud Maillard, Sébastien Assié, Aizhen Guo, Christine Citti, Eric Baranowski

**Affiliations:** ^1^The State Key Laboratory of Agricultural Microbiology, College of Veterinary Medicine, Huazhong Agricultural University, Wuhan, China; ^2^Hubei International Scientific and Technological Cooperation Base of Veterinary Epidemiology, International Research Center for Animal Disease, Ministry of Science and Technology of China, Wuhan, China; ^3^Key Laboratory of Preventive Veterinary Medicine in Hubei Province, The Cooperative Innovation Center for Sustainable Pig Production, Wuhan, China; ^4^Key Laboratory of Development of Veterinary Diagnostic Products, Key Laboratory of Ruminant Bio-products, Ministry of Agriculture and Rural Affairs of China, Wuhan, China; ^5^IHAP, ENVT, INRA, Université de Toulouse, Toulouse, France; ^6^BioEpAR, INRA, Oniris, Nantes, France

**Keywords:** *Mycoplasmas bovis*, extracellular DNA, cytotoxicity, hydrogen peroxide, pyruvate metabolism, virulence factors

## Abstract

Microbial access to host nutrients is a key factor of the host-pathogen interplay. With their nearly minimal genome, wall-less bacteria of the class *Mollicutes* have limited metabolic capacities and largely depend on host nutrients for their survival. Despite these limitations, host-restricted mycoplasmas are widely distributed in nature and many species are pathogenic for humans and animals. Yet, only partial information is available regarding the mechanisms evolved by these minimal pathogens to meet their nutrients and the contribution of these mechanisms to virulence. By using the ruminant pathogen *Mycoplasma bovis* as a model system, extracellular DNA (eDNA) was identified as a limiting nutrient for mycoplasma proliferation under cell culture conditions. Remarkably, the growth-promoting effect induced by supplementation with eDNA was associated with important cytotoxicity for actively dividing host cells, but not confluent monolayers. To identify biological functions mediating *M. bovis* cytotoxicity, we produced a library of transposon knockout mutants and identified three critical genomic regions whose disruption was associated with a non-cytopathic phenotype. The coding sequences (CDS) disrupted in these regions pointed towards pyruvate metabolism as contributing to *M. bovis* cytotoxicity. Hydrogen peroxide was found responsible for eDNA-mediated *M. bovis* cytotoxicity, and non-cytopathic mutants were unable to produce this toxic metabolic compound. In our experimental conditions, no contact between *M. bovis* and host cells was required for cytotoxicity. Further analyses revealed important intra-species differences in eDNA-mediated cytotoxicity and H_2_O_2_ production, with some strains displaying a cytopathic phenotype despite no H_2_O_2_ production. Interestingly, the genome of strains PG45 and HB0801 were characterized by the occurrence of insertion sequences (IS) at close proximity to several CDSs found disrupted in non-cytopathic mutants. Since PG45 and HB0801 produced no or limited amount of H_2_O_2_, IS-elements might influence H_2_O_2_ production in *M. bovis*. These results confirm the multifaceted role of eDNA in microbial communities and further identify this ubiquitous material as a nutritional trigger of *M. bovis* cytotoxicity. *M. bovis* may thus take advantage of the multiple sources of eDNA *in vivo* to modulate its interaction with host cells, a way for this minimal pathogen to overcome its limited coding capacity.

## Introduction

Beyond its role as universal support of genetic information, DNA is emerging as a multifaceted macromolecule with broad implications for microbial physiology and host defenses (Vorkapic et al., 2016; Gallucci and Maffei, 2017; Ibáñez de Aldecoa et al., 2017; Nagler et al., 2018). These novel properties are mainly attributed to extracellular DNA (eDNA), a ubiquitous material of prokaryotic and eukaryotic origin that can be isolated from a wide range of natural environments including biological fluids (Vlassov et al., 2007; Vorkapic et al., 2016; Aucamp et al., 2018; Nagler et al., 2018).

As a source of genetic information, eDNA contributes to bacterial genome maintenance and evolution (Mell and Redfield, 2014; Blokesch, 2017). Naturally competent bacteria are able to actively transport eDNA fragments across their cell envelope. This incoming genetic information can be used to repair damaged DNA, delete selfish mobile genetic elements, or acquire new phenotypic traits. As a biopolymer, eDNA is a structural component of biofilms (Das et al., 2013; Okshevsky and Meyer, 2015; Vorkapic et al., 2016; Ibáñez de Aldecoa et al., 2017; Nagler et al., 2018). Another important aspect of eDNA is its function as a source of nutrients (Vorkapic et al., 2016; Ibáñez de Aldecoa et al., 2017). Several bacteria use extracellular nucleases to degrade eDNA, whereas others use competence genes, or competence gene homologs, to uptake eDNA under nutritional stress.

Mycoplasmas are members of the class *Mollicutes*, a large group of wall-less bacteria that are well known for having some of the smallest genomes thus far characterized in free-living organisms (Citti and Blanchard, 2013). As such, mycoplasmas lack a significant number of biological functions found in more classical bacteria and have important nutritional requirements (Razin et al., 1998; Pereyre et al., 2009; Masukagami et al., 2017). Despite these limitations, mycoplasmas are widely distributed in nature where they live in close association with their hosts, with many species being successful human or animal pathogens. The control of these organisms is facing an alarming rate of drug resistance and a lack of effective vaccines causing growing concerns both in the medical and veterinary fields (Citti and Blanchard, 2013; Gautier-Bouchardon, 2018; Kirby, 2018). Among those, *Mycoplasma bovis* is emerging worldwide as a major cause of pneumonia and mastitis in cattle and is an important economic burden for the livestock industry (Bürki et al., 2015; Lysnyansky and Ayling, 2016; Nicholas and Ayling, 2016; Perez-Casal et al., 2017).

Mycoplasmas are recurrently found associated with cultures of mammalian cells, where they can survive for long periods, often without apparent signs of contamination. Yet, several pathogenic species are able to induce cellular vacuolization or a pronounced cytopathic effect (Rottem, 2003; Kannan and Baseman, 2006; Hegde et al., 2016; Josi et al., 2018). However, factors contributing to host cell damages are still poorly understood. The main reason is that mycoplasmas are lacking the classical repertoire of virulence genes found in more complex bacteria. Indeed, the only example of mycoplasma products exhibiting classical toxin-like activities is the ADP-ribosylating and vacuolating cytotoxin of the human pathogen *Mycoplasma pneumoniae* (Kannan and Baseman, 2006). The development of axenic and cell culture conditions together with the construction of specific tools for their genetic manipulation has greatly facilitated the study of these fastidious organisms. These advances have largely contributed not only to a better understanding of the remarkable plasticity of their surface architecture (Citti et al., 2010) but also to the discovery of massive horizontal gene transfers within and across species (Citti et al., 2018).

Despite their complex nutritional requirement, the mechanisms evolved by mycoplasmas to access nutrients essential for their multiplication *in vivo* are poorly understood. Nucleic acids are long known for influencing the proliferation of several fastidious mycoplasma species under axenic growth conditions (Razin and Knight, 1960). However, the role of eDNA in the infectious process of pathogenic species has never been addressed. In the present study, eDNA was identified as a limiting nutrient for mycoplasma proliferation in cell culture conditions, using the ruminant pathogen *Mycoplasma bovis* as a model system. The growth-promoting effect of eDNA on *M. bovis* was associated with important cytotoxicity for actively dividing cells, an event mediated by the release of H_2_O_2_ in the culture medium.

## Materials and Methods

### Mycoplasmas, Cell Lines, and Culture Conditions

*M. bovis* strains and colony-purified isolates used in the present study are described in Table 1. Mycoplasmas were grown at 37°C in SP4 medium supplemented with 45 μg/ml cefquinome (Cobactan, MSD Animal Health). Gentamicin (50 μg/ml) was added to the media for the propagation of *M. bovis* mutants generated by transposon mutagenesis. Mycoplasma titers were determined based on colony counts on solid media after 3–5 days of incubation at 37°C. The embryonic bovine lung (EBL) cell line was kindly provided by M. Heller (Friedrich-Loeffler-Institute, Jena, Germany). Cells were grown in DMEM-based medium consisting of Dulbecco’s modified Eagle’s medium (DMEM, high glucose, sodium pyruvate, and GlutaMAX-I; Gibco) supplemented with non-essential amino acids (NEAA, Gibco) and 10% heat-inactivated fetal bovine serum (FBS, Gibco). The mycoplasma-free status of the cell line was tested by using a genus-specific PCR (van Kuppeveld et al., 1992).

**Table 1:** *M. bovis* type and field strains used in the present study.

Strain^a^	Origin	Year of isolation	Host	Clinical manifestations	Reference
PG45	USA	1962	Bovine	Mastitis	Wise et al. (2011)
HB0801	China	2008	Bovine	Respiratory	Qi et al. (2012)
HB0801 P150	China	2008	Bovine	Respiratory	Zhang et al. (2014)
RM16	France	2016	Bovine	Respiratory	This study
1067	France	1983	Bovine	Mastitis	Le Grand et al. (2002)
7103	France	2016	Bovine	Respiratory	This study
4785	France	1990	unknown	Respiratory	Marenda et al. (2005)
8790	Ethiopia	1987	Caprine	Respiratory	Manso-Silván et al. (2012)
SA1	France	2004	Bovine	Respiratory	This study
SA2	France	2004	Bovine	Respiratory	This study
SA5	France	2005	Bovine	Respiratory	This study
SA7	France	2006	Bovine	Respiratory	This study

a *M. bovis strain HB0801 P150 was selected by 150 serial passages of the virulent strain HB0801. Whole-genome sequences of strains PG45, HB0801, and HB0801 P150 are available in databanks with accession numbers NC_014760.1/CP002188.1, NC_018077.1/CP002058.1, and NZ_CP007590.1/CP007590.1, respectively. Draft genome sequences of strains 1067 and 8790 are available as whole-genome shotgun projects with accession numbers LAUT01 and LAUS01, respectively. RM16, SA1, SA2, SA5, SA7 are colony-purified isolates originating from the French department number 15 (Cantal; RM16), 44 (Loire-Atlantique; SA1, SA2, SA7), and 85 (Vendée; SA5). SA1 and SA2 were isolated from the same animal (SA1, transtracheal aspirate; SA2, lung sample). These isolates were originaly named as 415 MAE 04 (SA1 and SA2), 413 MAE 05 (SA5), and 453 MAE 06 (SA7)*.

### Co-cultivation of *M. bovis* With Bovine Lung Cells and Crystal Violet Cell Cytotoxicity Assay

*M. bovis* and EBL cells were co-cultivated in DMEM-based medium, as previously described (Baranowski et al., 2010). Briefly, EBL cells were seeded in 24-well plates (Falcon) at a density of 5 × 10^3^ cells/cm^2^ and inoculated with *M. bovis* at multiplicity of infection (MOI) of 2. Since SP4 medium (up to 0.1%) has no apparent toxic effect on EBL cells (data not shown), *M. bovis* inoculums were prepared by direct dilution of mycoplasma stock cultures with predefined titers in DMEM-based medium. At different time post-inoculation, mycoplasma titers were determined by CFU titration following one freeze-thaw cycle. Mycoplasma cytotoxicity was determined by staining cell monolayers with crystal violet. Cell monolayers were washed with Dulbecco’s phosphate-buffered saline (DPBS, Gibco) and stained for 60 min with 0.1% crystal violet solution in 10% ethanol. After washing with water, plates were air-dried and incubated with 95% ethanol for 2 h at room temperature before measuring the optical density at 590 nm.

To test the influence of extracellular nucleic acids on *M. bovis* cytotoxicity, mycoplasmas and EBL cells were co-cultivated in DMEM-based medium supplemented with increasing concentration of calf thymus DNA (UltraPure^™^ Calf Thymus DNA Solution, Invitrogen) and/or yeast tRNA (Invitrogen). Calf thymus DNA treatment with Proteinase K (Qiagen), DNase I (Invitrogen), and RNase A (Invitrogen) was performed by 1 h incubation in DMEM medium and 15 min heating at 96°C. Polynucleotides were removed from DNase I digestion products by using the QIAquick PCR Purification Kit (Qiagen).

To test the cytotoxicity of cell culture supernatants infected with *M. bovis*, mycoplasma cells were killed by a gentamicin treatment (400 μg/ml) for 3 h at 37°C and mycoplasma cells were depleted by 30 min centrifugation at 12,000 ×*g* (4°C). These treated supernatants were then applied to EBL cells (5 × 10^3^ cells/cm^2^) for 72 h, and cytotoxicity was measured by crystal violet staining. Catalase from bovine liver (Sigma) was used to remove H_2_O_2_ from cell culture supernatants (60 U/ml; 2 h incubation at room temperature).

### Random Transposon Mutagenesis in *M. bovis*

Transposon mutagenesis was carried out by using plasmid pMT85, as previously described (Baranowski et al., 2010). Plasmid pMT85, which contains a modified version of Tn*4001* (mTn) was kindly provided by Richard Herrmann (Zimmerman and Herrmann, 2005). Briefly, gentamicin-resistant colonies were collected from 18 independent transformations and grown in 1 ml of selective SP4 medium. Individual mutants were distributed in 96-well plates and stored at −80°C. Transposon insertion sites in the *M. bovis* chromosome were mapped by sequencing the junction between mycoplasma genomic DNA and the mTn. Briefly, *M. bovis* mutants were amplified by single-primer PCR (Karlyshev et al., 2000) using oligonucleotide primers SG6pMT85 (CGGAAACCAGGCAACGAC) or SG8pMT85 (GAGTCAGTGAGCGAGGAAGC) and the following annealing temperatures: 55 (20 cycles), 30 (30 cycles), and 55°C (30 cycles). The PCR products were sequenced with the nested primers SG6-2 (GCCGCGTCAATTGGTGGGGT), SG6-3 (ACGGCCGATGAATCCTCTA) or EB8 (GGAAGAGCGCCCAATACGCA) (Eurofins Genomics).

### Identification of *M. bovis* Mutants With Reduced Cytotoxicity

*M. bovis* mutants displaying reduced cytotoxicity for EBL cells were selected from the mutant library by high-throughput screening in 96-well plates (Falcon). EBL cells were seeded at a density of 5 × 10^3^ cells/cm^2^ in DMEM-based medium supplemented with 10 μg/ml calf thymus DNA (Invitrogen) and inoculated with individual mutants using the 96-pin replicator (Boekel Scientific). After 3 days of co-cultivation, cells were stained with crystal violet (see above) and *M. bovis* mutants displaying reduced cytotoxicity were selected by visualization of the cytopathic effect. The non-cytotoxic phenotype of the selected mutants was confirmed by a second co-cultivation with EBL cells in larger 24-well plates (Falcon).

### Quantification of Hydrogen Peroxide Production

The production of H_2_O_2_ by *M. bovis* was estimated by using MQuant^™^ Peroxid-test strips (Merck), and the H_2_O_2_ concentration was determined by using the MyQubit Amplex^™^ Red Peroxide Assay (Invitrogen). Both assays were performed according to the manufacturer’s recommendations.

### Illumina Whole-Genome Sequencing, Bioinformatic Analyses

The genome of *M. bovis* RM16 was sequenced using HiseqIllumina technology (paired-end, 2 × 150 bp, with an average of 3,000 X for coverage depth; Illumina, San Diego, CA) at the GATC Biotech facility (Konstanz, Germany). Bioinformatics analyses were conducted using the GenoToul Bioinformatics facility, Toulouse, France. Quality of the sequencing reads was controlled with FASTQC tool.^1^ *De novo* assembly was done using Abyss v2.1.5 (Simpson et al., 2009) and resulted in 61 contigs. The RM16 genome was annotated using the RAST pipeline (Overbeek et al., 2014). Co-linear analysis of the RM16 sequence with the genome of PG45 and HB0801 strain was performed using MUMmer 3.0 or ACT 13.0.0 software (Kurtz et al., 2004; Carver et al., 2005). The Whole Genome Shotgun project of *M. bovis* strain RM16 has been deposited at DDBJ/ENA/GenBank under the accession VSDF00000000. The version described in this paper is version VSDF01000000. Sequences of interest were compared for similar nucleotide sequences with BLAST-NCBI search utility.^2^

## Results

### Extracellular DNA Is a Limiting Nutrient for *M. bovis* Proliferation in Cell Culture

**Figure 1:**
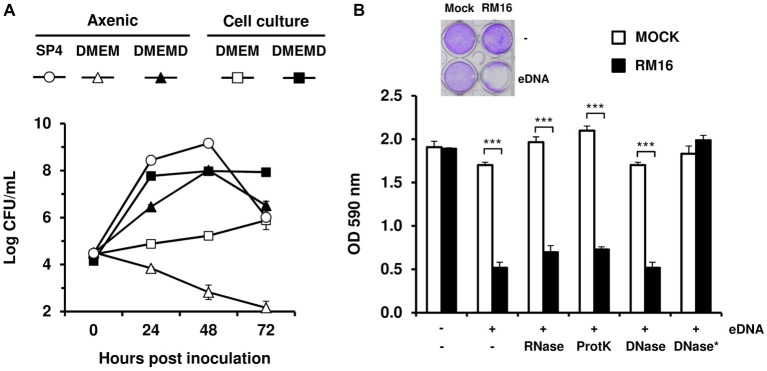
The growth-promoting effect of eDNA on *M. bovis*. Comparative growth of *M. bovis* under axenic and cell culture conditions **(A)**. RM16 proliferation was monitored in SP4 medium (SP4), cell culture medium (DMEM), and cell culture medium supplemented with 10 μg/ml calf thymus DNA (DMEMD). Mycoplasma titers (log CFU/ml) were determined by CFU titrations. The cytopathic effect induced by *M. bovis* upon co-incubation with host cells **(B)**. EBL cells (10^4^ cells) were inoculated with RM16 at an MOI of 2 (RM16) or mock-infected (Mock). After 72 h of co-incubation, monolayers were stained with crystal violet and survival cells were estimated by measuring the optical density at 590 nm (OD 590). When indicated, DMEM-based medium was supplemented with 10 μg/ml calf thymus DNA (eDNA). Calf thymus DNA was subjected to the following enzymatic treatments: RNase A (RNase), Proteinase K (ProtK) and DNase I (DNase) digestion (see section “Materials and Methods”). The asterisk indicates that polynucleotides were removed from DNase I digestion products (DNase*). Infected and mock-infected samples were treated identically. Data are the means of at least three independent assays. Standard deviations are indicated by error bars. *p*-values were determined by using two-sided independent sample *t* tests and comparing OD590 values of RM16-infected samples to those of mock-infected samples (^***^p < 0.001).

Transtracheal aspirates from animals showing signs of bronchopneumonia were used to isolate *M. bovis* RM16 (Table 1). This field isolate, whose draft genome sequence was determined by illumina sequencing, was used as a low-passage strain to investigate the influence of eDNA on *M. bovis* proliferation upon co-cultivation with embryonic bovine lung (EBL) cells. RM16 and EBL cells were seeded in DMEM-based medium at a MOI of 2, and mycoplasma growth was monitored by enumerating CFUs over a period of 72 h (Figure 1A). Data showed that EBL cells were required for RM16 proliferation, and DMEM-based medium alone was unable to sustain mycoplasma growth. However, EBL cells had only a limited effect on RM16 growth and maximum mycoplasma titers were about 10^3^-fold lower than those yielded in SP4, a medium commonly used for the propagation of mycoplasmas under axenic conditions. Remarkably, DMEM-based medium supplementation with calf thymus DNA (10 μg/ml) was found to dramatically enhance the proliferation of RM16 both under axenic and cell culture conditions. This growth-promoting effect was associated with important cytotoxicity for EBL cells leading to a complete cytopathic effect (CPE) after 72 h of co-incubation (Figure 1B). Incubation of EBL cells with calf thymus DNA alone failed to reveal any CPE ruling out any possible toxicity of the DNA solution. Finally, EBL cell density was identified as critical for RM16 cytotoxicity, since it was only observed upon *M. bovis* co-incubation with actively dividing cells (Supplementary Figure S1A).

To further demonstrate that eDNA is a limiting nutrient for the propagation of *M. bovis* in cell culture and its cytotoxicity for EBL cells, calf thymus DNA was subjected to different enzymatic digestions (Figure 1B). While RNase A and proteinase K had no influence, DNase I was able to abrogate the cytopathic growth of RM16 after removal of small polynucleotides generated by DNA digestion. This confirms that *M. bovis* cytotoxicity for EBL cells is induced by the growth-promoting effect of eDNA and further suggests that *M. bovis* can take the advantage of extracellular polynucleotides for its proliferation in cell culture. Nucleic acid requirements of *M. bovis* were further investigated by evaluating the CPE induced by RM16 proliferation. The DNA concentration needed to reach 50% CPE in 72 h was estimated at about 5 μg/ml using DNA of eukaryotic (calf thymus) or prokaryotic (*Escherichia coli* K-12 DH10B) origin. Yeast tRNAs, alone or in combination with calf thymus DNA, had only a limited influence on the cytotoxicity of RM16 for EBL cells (Supplementary Figure S1B).

These results clearly identify eDNA as a nutritional trigger of the *M. bovis* cytopathic effect on cultured cells.

### *M. bovis* Cytotoxicity Is Linked to Pyruvate Metabolism

**Figure 2:**
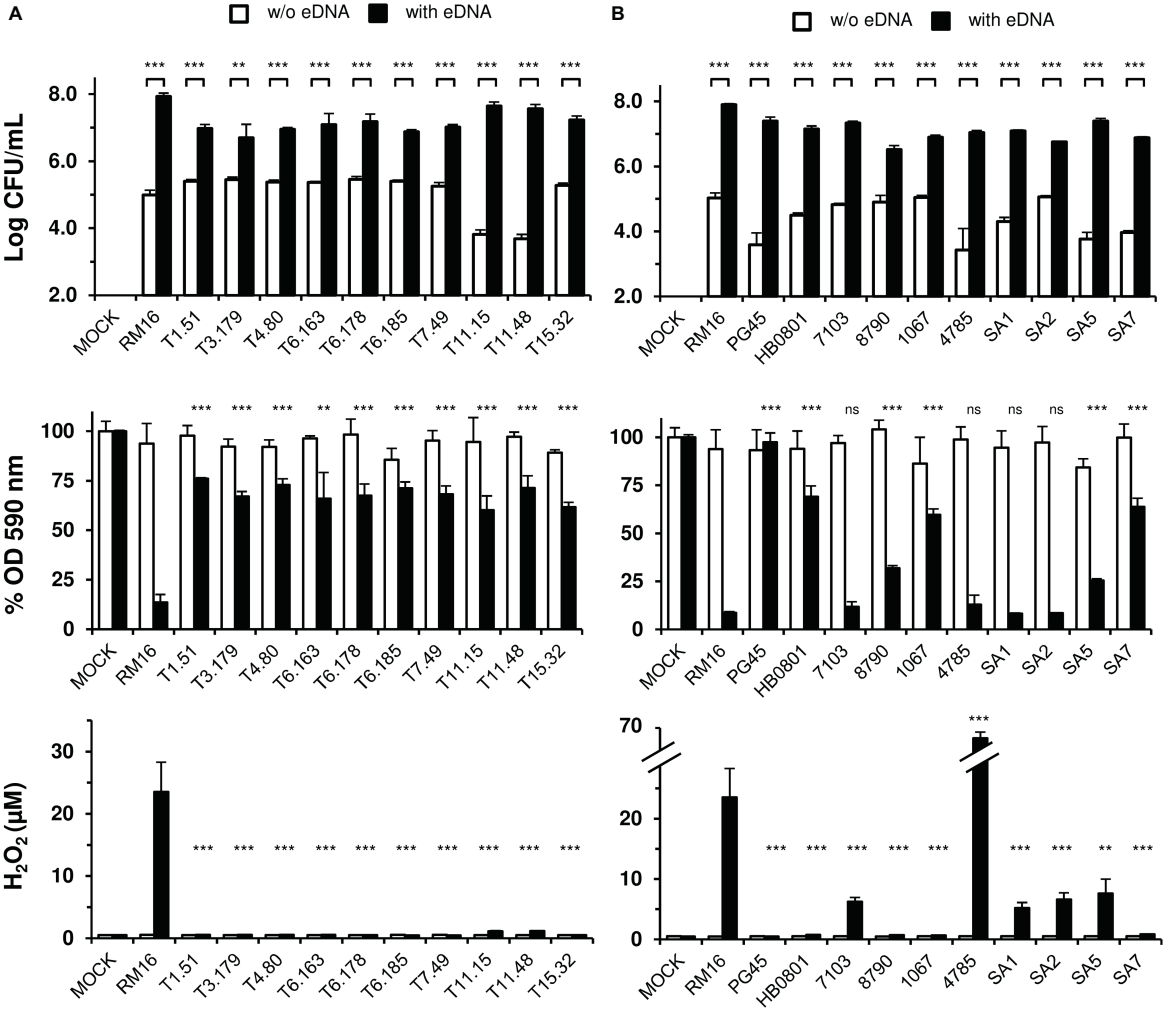
Phenotypic characterization of *M. bovis* knockout mutants **(A)** and *M. bovis* type and field strains **(B)**. *M. bovis* mutants and strains are described in Tables 1, 2. Mycoplasmas were co-incubated with EBL cells in DMEM-based medium supplemented with 10 μg/ml calf thymus DNA (black bars) or DMEM-based medium without DNA (white bars). EBL cells (10^4^ cells) were inoculated at an MOI of 2. After 72 h of co-incubation, samples were used either for CFU titrations (log CFU/ml), analysis of EBL cell survival (% OD590 nm) or hydrogen peroxide production (H_2_O_2_). Infected and mock-infected (Mock) samples were treated identically. Data are the means of at least three independent assays. Standard deviations are indicated by error bars. *p*-values were determined by using two-sided independent sample *t* tests and comparing (1) mycoplasma titers (log CFU/ml) reached in the presence of DNA to those reached without supplementation, and (2) EBL cell survival (% OD590 nm) or (3) H2O2 production (H2O2) following co-incubation with *M. bovis* mutants or strains to values obtained with RM16 (ns, *p* > 0.05; ^**^p < 0.01; ^***^p < 0.001).

**Figure 3:**
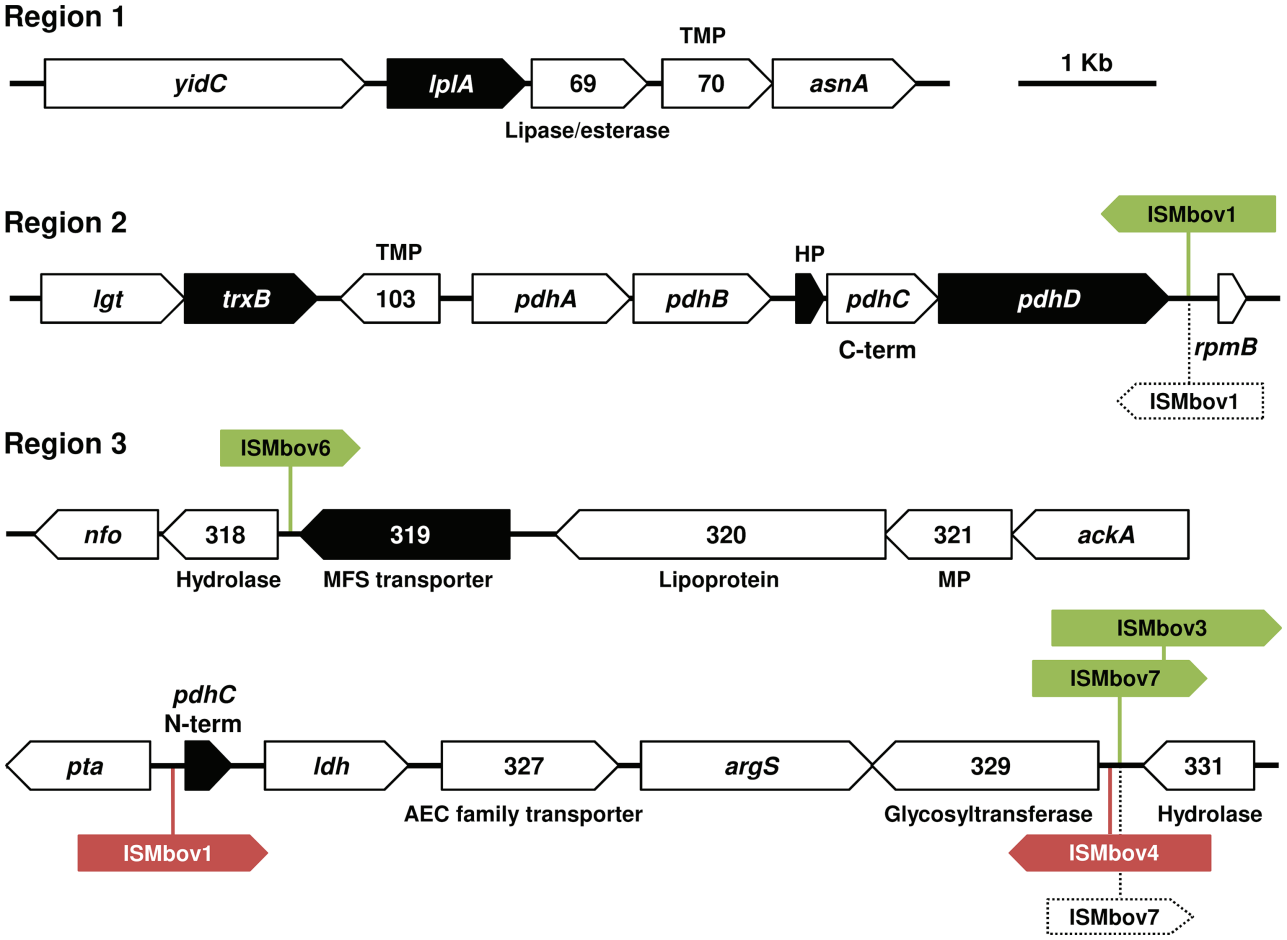
Analysis of *M. bovis* loci in non-cytopathic mutants. For each locus, CDS found disrupted in non-cytopathic mutants are indicated by black arrows (Table 2) and surrounding CDS by open arrows. The CDS annotations are indicated either by the gene name or the MBOVPG45 locus tag number. Putative functions or features of CDS products are indicated when available: MP, membrane protein; HP, hypothetical proteins; Lipo, lipoproteins. Green and red arrows indicate the position and name of IS transposase in the genome of *M. bovis* strains HB0801 and PG45, respectively. The occurrence of IS-elements in the draft genome sequence of RM16 was suggested by sequence homology at contig ends. The position of these IS-elements in RM16 is are indicated by dotted boxes.

To identify genomic regions that are critical for *M. bovis* cytotoxicity, a library of 3,072 individual mutants was generated by transposon mutagenesis in RM16, and non-cytopathic mutants were selected by high-throughput screening in cell culture. Transposon mutagenesis was performed by using a modified version of Tn*4001* (mTn). Because mTn contains a gentamicin-resistance marker but no transposase gene, its random insertion in the mycoplasma genome is stable in addition to conferring antibiotic resistance. By using this screening strategy, 13 non-cytopathic mutants were selected that revealed 12 unique mTn insertion sites distributed within eight coding sequences (CDS) (Table 2). The two CDS found disrupted in mutants T1.77, T14.18, and T15.101 (Table 2) were consistent with our previous studies that identified *apt* and the *sufS-sufU* locus as essential for the proliferation of *Mycoplasma agalactiae* in cell culture (Baranowski et al., 2010; Skapski et al., 2011). These three mutants were not further characterized since their non-cytopathic phenotype was associated with a severe growth deficiency in cell culture (data not shown). The non-cytopathic mutants, whose proliferation in cell culture produced CFU titers similar to RM16 (Figure 2A), were characterized by a transposon inserted within six CDS located at three distant chromosomal regions (Figure 3). Remarkably, several CDS found disrupted in these distant regions were homologous to components of the pyruvate dehydrogenase (PDH) complex or were located in close proximity to this gene cluster (Figure 3 and Table 2). These included (1) a *pdhD* homolog (mutant T6.185), (2) a CDS sharing similarity with the N-terminus of *pdhC*, which is located upstream of the lactate dehydrogenase homolog in RM16 (mutant T07.49), (3) a CDS encoding a hypothetical protein with unknown function located in front of the C-terminus of *pdhC* (mutant T01.051), and (4) a CDS predicted to encode a lipoate protein ligase that catalyzes the transfer of lipoic acid to lipoate-dependent enzymes, such as the lipoamide dehydrogenase of the PDH complex (mutants T3.179 and T4.80). Finally, two additional regions were found repeatedly disrupted in non-cytopathic mutants. These included a thioredoxin reductase TrxB gene homolog (mutants T11.15 and T11.48) and a CDS predicted to encode a putative membrane protein belonging to the major facilitator superfamily (MFS), a family of permease that facilitates the movement of small solutes across cell membranes in response to chemiosmotic ion gradients (mutants T15.32, T6.163, and T6.178).

**Table 2:** Transposon insertion sites in the chromosome of non-cytopathic *M. bovis* mutants.

Genomic position^a^ (orientation)	Mutant^b^	CDS in PG45^c^	CDS position^d^	CDS identity
077424 (+)	T03.179	MBOVPG45_ 0068	0.12 (+)	Lipoate-protein ligase *(lplA)*
077896 (+)	T04.080	MBOVPG45_ 0068	0.49 (+)	Lipoate-protein ligase (*lplA*)
094369 (−)	T14.018	MBOVPG45_ 0082	0.03 (−)	Iron–sulfur cluster assembly scaffold protein (*sufU*)
110078 (−)	T11.015	MBOVPG45_ 0102	0.26 (−)	Thioredoxin-disulfide reductase (*trxB*)
110078 (−)	T11.048	MBOVPG45_ 0102	0.26 (−)	Thioredoxin-disulfide reductase (*trxB*)
114118 (+)	T01.051	MBOVPG45_ 0106	0.43 (+)	HP upstream of a truncated form of the dihydrolipoamide acetyltransferase (*pdhC*), E2 component of the pyruvate dehydrogenase complex (PDHC)
115426 (−)	T06.185	MBOVPG45_ 0108	0.28 (−)	Dihydrolipoamide dehydrogenase (*pdhD*), E3 component of the PDHC
359741 (−)	T15.032	MBOVPG45_ 0319	0.51 (+)	Membrane protein, major facilitator superfamily (MFS) transporter
360195 (+)	T06.163	MBOVPG45_ 0319	0.20 (−)	Membrane protein, major facilitator superfamily (MFS) transporter
360451 (−)	T06.178	MBOVPG45_ 0319	0.03 (+)	Membrane protein, major facilitator superfamily (MFS) transporter
368231 (+)	T07.049	MBOVPG45_ 0325	0.82 (+)	Biotin/lipoyl-binding protein similar to the N-terminus of the dihydrolipoamide acetyltransferase (*pdhC*)
918,741 (+)	T01.077	MBOVPG45_ 0796	0.60 (−)	Adenine phosphoribosyltransferase (*apt*)
918785 (−)	T15.101	MBOVPG45_0796	0.51 (+)	Adenine phosphoribosyltransferase (*apt*)

a *The position of the mTn insertion was defined based on the PG45 published sequence in GenBank database (CP002188.1). The orientation of the mTn is given in parenthesis using the gentamicin-resistance marker as a reference. The mTn in T11.15 and T11.48 was inserted at the same chromosomal position*.

b *Mutants were designated according to transformation and clone numbers (e.g., T03.179 designates clone 179 isolated from transformation T03)*.

c *The CDS found disrupted in *M. bovis* are indicated by their mnemonic codification in GenBank*.

d *For each CDS, the relative position and orientation of the mTn are indicated*.

These data suggest that housekeeping functions linked to pyruvate metabolism may influence the cytotoxicity of *M. bovis* in cell culture.

### Hydrogen Peroxide Production by *M. bovis* Is Cytotoxic for Embryonic Bovine Lung Cells

The PDH is a key enzymatic complex linking glycolysis to citric acid cycle. Under aerobic conditions, this complex catalyzes the conversion of pyruvate into acetyl-CoA and produces NADH. In *M. bovis*, the NAD^+^ pool can be regenerated by an H_2_O_2_-producing NADH oxidase (Zhao et al., 2017). This prompted us to examine whether *M. bovis* was able to produce H_2_O_2_ under cell culture conditions (Figure 2A). Interestingly, robust production of H_2_O_2_ reaching up to 30 μM was detected for RM16, whereas non-cytopathic mutants produced undetectable levels of H_2_O_2_. As expected, H_2_O_2_ production by RM16 was conditioned by the presence of calf thymus DNA (Figure 2A). Since defense mechanisms developed by mammalian cells against oxidative stress may decrease the amount of H_2_O_2_ produced by *M. bovis*, we reexamined the capacity of RM16 and non-cytopathic mutants to produce H_2_O_2_ in axenic conditions using DMEM-based medium supplemented with eDNA (Supplementary Figure S2). The composition of the DMEM-based medium was also modified by removing the pyruvate, which is well known for having H_2_O_2_-scavenging properties. In these conditions, RM16 was found to produce higher levels of H_2_O_2_ reaching up to 75 μM and two non-cytopathic mutants, namely T11.15 and T11.48, produced about 25 μM of H_2_O_2_. Surprisingly, these two mutants were characterized by a mTn insertion in *trxB* whose function is usually to protect bacteria from the toxic effects of H_2_O_2_ (Lu and Holmgren, 2014). Interestingly, *trxB* is located a few kps upstream of the PHD locus (*pdhA*, *pdhB*, *pdhC*, and *pdhD*) (Figure 3). Whether the phenotype observed in T11.15 and T11.48 is due to *trxB* inactivation or to a polar effect of the mTn insertion on the PHD locus remains to be elucidated.

The cytotoxicity of H_2_O_2_ has been extensively studied in several mycoplasma species (Blötz and Stülke, 2017). In the ruminant pathogen *Mycoplasma mycoides* subsp. *mycoides*, cytotoxicity was associated with a glycerol-3-phosphate oxidase GlpO found at the mycoplasma membrane surface suggesting that close contact to the host cell is required (Pilo et al., 2005). In RM16, the production of H_2_O_2_ was found independent of glycerol metabolism since cell culture medium supplementation with glycerol (100 μM) failed to induce detectable production of H_2_O_2_ (data not shown). This result was consistent with the lack of a *glpO* homolog in RM16 and whole-genome sequences of *M. bovis* available in databases. Another particular feature of RM16 is that cytotoxicity was not dependent on close contact between bacteria and EBL cells since the amount of H_2_O_2_ released by RM16 in the cell culture medium can induce cytotoxicity. This was shown by using gentamicin-treated cell culture supernatants collected after 72 h of co-incubation with RM16 (Supplementary Figure S1C). Indeed, these bacteria-free supernatants were found cytotoxic for EBL cells. Catalase (60 U/ml) was shown to abolish the cytotoxicity of the supernatants discarding any possible cytotoxic effect due to the depletion of nutrients.

These results clearly identified H_2_O_2_ as responsible for the cytotoxicity of RM16 and further suggest that this toxic metabolic compound may be a common virulence factor in ruminant mycoplasma species, although several different metabolic pathways may be involved.

### Intra-species Differences in eDNA-Mediated *M. bovis* Cytotoxicity and H_2_O_2_ Production

The CDS organization of the three genomic regions associated with H_2_O_2_ production in RM16 was found highly conserved in *M. bovis* strains PG45 and HB0801, and only differ by the presence of several insertion sequences (IS) elements (Figure 3). However, previous studies reported important intra-species differences in H_2_O_2_ production (Khan et al., 2005). This led us to further characterize the cytopathic properties of *M. bovis* using a collection of type and field strains (Table 1). As expected, eDNA was found to have a dramatic effect on their proliferation with mycoplasma titers reaching values ranging from 3.4 × 10^6^ to 7.9 × 10^7^ CFU/ml after 72 h of co-incubation with EBL cells (Figure 2B). Interestingly, staining with crystal violet revealed important differences in the survival of EBL cells, with strains RM16, 7103, 4785, SA1, SA2, and SA5 displaying survival rates lower than 25% when compared to mock-infected EBL cells, and strain PG45 having no cytotoxicity. Strains HB0801, 8790, 1067, and SA7 were characterized by an intermediate level of cytotoxicity with survival rates ranging from 25 to 75%. These differences showed no obvious correlation with mycoplasma titers (Figure 2B). For instance, the survival rate associated with strain 8790 was 32% with a mycoplasma titer of 3.4 × 10^6^ CFU/ml, while the non-cytopathic strain PG45 reached up to 2.6 × 10^7^ CFU/ml. Important differences were also observed in the amount of H_2_O_2_ measured after 72 h of co-incubation with EBL cells (Figure 2B). Interestingly, *M. bovis* strains producing undetectable levels of H_2_O_2_ were found poorly cytopathic, with survival rates higher than 50%. Conversely, survival rates lower than 50% were associated with the production of 5–68 μM of H_2_O_2_. One exception was 8790, an atypical strain of *M. bovis* isolated from caprine that displayed an intermediate level on cytotoxicity (32%) with no production of H_2_O_2_. As observed for RM16, *M. bovis* strains tested were only found cytotoxic for actively dividing EBL cells but not confluent monolayers (Supplementary Figure S1A).

Whether mobile IS-elements may influence *M. bovis* production of H_2_O_2_ is unknown. However, the occurrence of these mobile elements at close proximity of several CDS found disrupted in RM16 non-cytopathic mutants provides a reasonable scenario for explaining the absence of H_2_O_2_ production by PG45 and HB0801. The recent release of *M. bovis* draft genome sequences in databases led us to further examine the influence of IS-elements on H_2_O_2_ production. The alignment of PG45 with the draft genome sequence of 1067 revealed several contigs ending at positions corresponding to IS-elements in PG45 suggesting a conserved organization at genomic regions associated with H_2_O_2_ production in these two strains that were found unable to produce detectable levels of this metabolic compound. In contrast, no IS-element was identified in the atypical strain 8790 suggesting that additional genomic regions may modulate H_2_O_2_ production in this distant phylogenic species.

**Figure 4:**
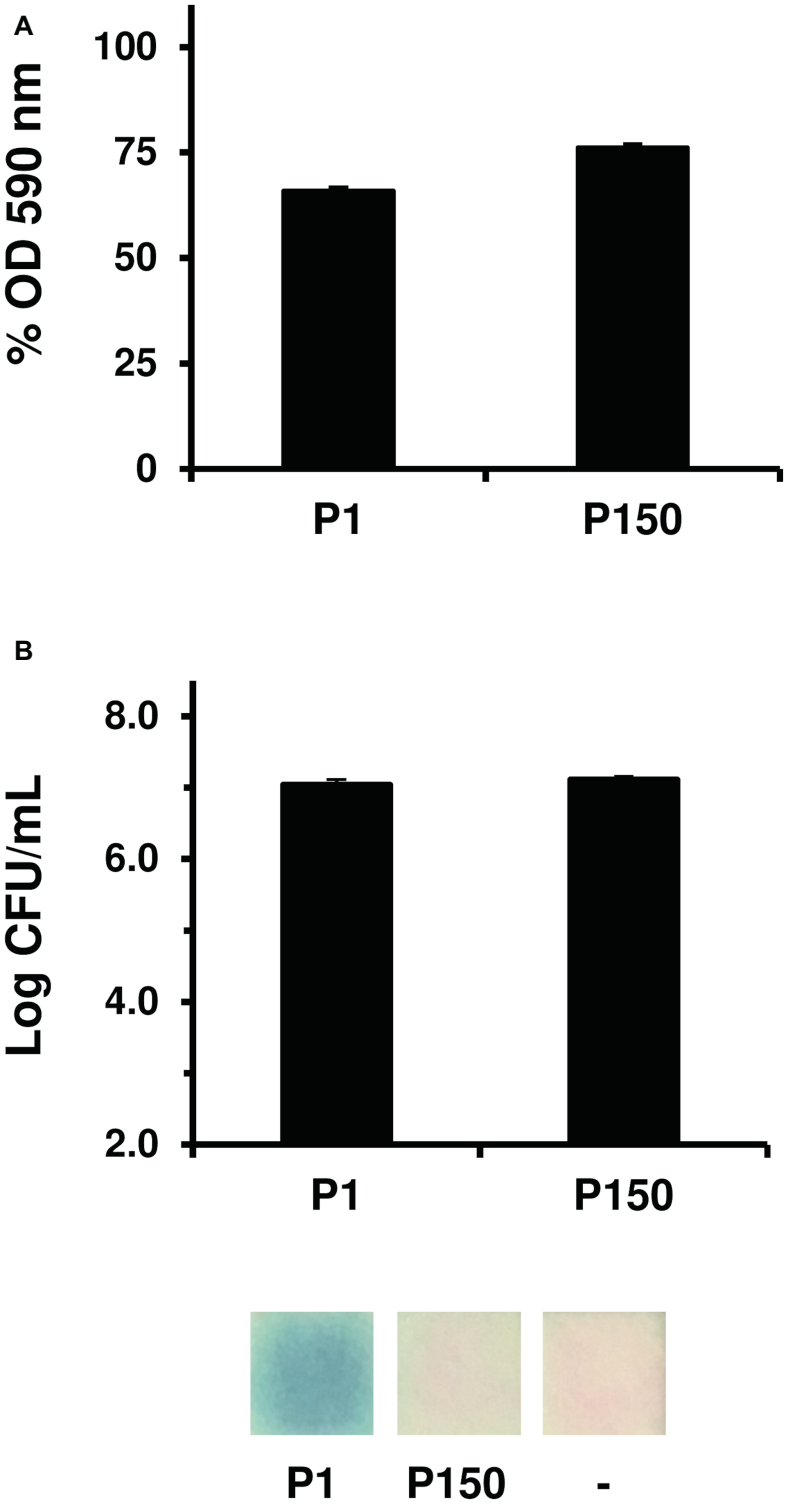
Phenotypic characterization of *M. bovis* vaccine strain P150. EBL cell survival (% OD590 nm) after 72 h of co-incubation with HB0801 (P1) and P150 (P150) in DMEM-based medium supplemented with 10 μg/ml calf thymus DNA **(A)**. Mycoplasma titers (log CFU/ml) reached by P1 and P150 after 48 h incubation in axenic DMEM-based medium without pyruvate and supplemented with 10 μg/ml calf thymus DNA and H_2_O_2_ production as determined by using MQuant^™^ Peroxid-test strips **(B)**. Data are the means of at least three independent assays. Standard deviations are indicated by error bars.

The cytotoxicity of *M. bovis* for EBL cells raised questions regarding the role of eDNA and H_2_O_2_ production on its virulence upon replication in the natural host. To address this issue, we have compared the cytotoxicity of the virulent strain HB0801 with its attenuated derivative P150 which was selected by 150 serial passages under axenic conditions (Zhang et al., 2014). Upon co-cultivation with EBL cells, HB0801 and P150 displayed intermediate levels of cytotoxicity with survival rates of about 50 and 75%, respectively (Figure 4A). As expected by our previous results, neither HB0801 nor P150 was able to produce detectable levels of H_2_O_2_ in cell culture, despite medium supplementation with calf thymus DNA. Since HB0801 and P150 were previously found to produce H_2_O_2_ (Rasheed et al., 2017), we re-examined their capacity to produce of H_2_O_2_ in axenic conditions using DMEM-based medium supplemented with eDNA (Figure 4B). In these conditions, HB0801 was found to produce detectable levels of H_2_O_2_ (estimated at 5–15 μM), whereas H_2_O_2_ production by P150 remained undetectable despite similar mycoplasma titers (Figure 4B). These results confirm that long-term serial propagation of HB0801 in axenic conditions led to the selection of variants with decreased production of H_2_O_2_ (Rasheed et al., 2017). The alignment of P150 with the genome of HB0801 identified a single nucleotide deletion in the CDS located upstream of *lplA* (Figure 3), which was found disrupted in RM16 non-cytopathic mutants T3.179 and T4.080 (Table 2). This deletion occurred in poly-A tract located in the central part of the CDS (genomic positions 76308-76315 in HB0801 and 76306-76312 in P150) leading to a truncation of its C-terminal region. Whether these changes are contributing to the attenuated phenotype of P150 is unknown, but they provide valuable information for the study of virulence factors in this pathogenic mycoplasma species.

Altogether, these data identify H_2_O_2_ production as contributing to the cytotoxicity of *M. bovis* under cell culture conditions and further suggest that additional mechanisms may also contribute to its cytopathogenicity.

## Discussion

Competition for nutrients is a fundamental aspect of host-microbe interactions. Despite important nutritional requirements, only limited information is available regarding the mechanisms that mycoplasmas have evolved to meet these requirements and the contribution of these mechanisms to virulence. Here, we showed that eDNA is a limiting nutrient for *M. bovis* proliferation upon co-cultivation with bovine lung cells. Indeed, medium supplementation with eDNA was found to dramatically enhance mycoplasma growth under cell culture conditions leading to cytopathic manifestations due to H_2_O_2_ production.

### The Pathometabolism of Minimal Bacteria

Studies with the ruminant pathogen *Mycoplasma mycoides* subsp. *mycoides* provided early evidence that carbon metabolism is intimately linked to pathogenicity in the mollicutes (Pilo et al., 2007; Blötz and Stülke, 2017). The genetic and biochemical characterization of glycerol uptake in two distinct phylogenetic lineages showing differences in their pathogenicity identified glycerol metabolism and H_2_O_2_ production as a potential virulence factor in this species (Vilei and Frey, 2001). In cell culture, the H_2_O_2_ producing glycerol-3-phosphate oxidase GlpO was shown to be responsible for the massive cytopathic effect induced by pathogenic strains of *M. mycoides* subsp. *mycoides* upon addition of glycerol in the culture medium (Pilo et al., 2005; Bischof et al., 2008). Remarkably, no GlpO homolog has been identified in *M. bovis* genome sequences available in databases, suggesting that this species has evolved glycerol-independent mechanisms of virulence (Bürki et al., 2015).

Recently, the hydrogen sulfide producing cysteine desulfurase/desulfhydrase HapE of *M. pneumoniae* was found to promote erythrocyte lysis and to alter the cytokine profile and growth of bronchial epithelial cells (Großhennig et al., 2016; Li et al., 2019b). Previously annotated as a cysteine desulfurase NifS/SufS, HapE is highly conserved across *Mollicutes* together with the iron-sulfur cluster assembly scaffold protein (SufU). Whether other pathogenic species are producing hydrogen sulfide is unknown, but the *sufS-sufU* locus in the ruminant pathogen *M. agalactiae* was found essential for survival in cell culture and colonization of the animal host (Baranowski et al., 2010, 2014; Skapski et al., 2011). The growth-deficient phenotype of mutant 14.018 (Table 2) confirmed the role of the *sufS-sufU* locus in *M. bovis* proliferation in cell culture. The close genetic proximity between *M. agalactiae* and *M. bovis* further suggests a possible role in host colonization.

Transposon mutagenesis in *M. bovis* suggests that glycerol-independent mechanisms of H_2_O_2_ production and cytotoxicity may be linked to pyruvate metabolism. Further studies are needed to confirm these results, but the release of cytotoxic metabolic compounds is emerging as pivotal in the pathometabolism of these minimal organisms. The release of H_2_O_2_ by RM16 in our experimental conditions was estimated at about 30 μM, a value 5-fold lower than those reported for the highly pathogenic *M. mycoides* subsp. *mycoides* pathogenic strain Afadé (Vilei and Frey, 2001; Pilo et al., 2005). Despite different experimental conditions, this result was consistent with the lower cytotoxicity of RM16 that was only observed upon co-incubation with actively dividing cells but not confluent monolayers. Since glycerol is metabolized into pyruvate under aerobic conditions, *M. mycoides* subsp. *mycoides* may take advantage of these two pathways to produce high levels of H_2_O_2_. While the NADH oxidase (Nox) is likely to be the main enzyme responsible for H_2_O_2_ production during pyruvate metabolism, both Nox and GlpO may be involved in glycerol metabolism. The *M. mycoides* subsp. *mycoides* GlpO (Pilo et al., 2005) and the *M. bovis* Nox (Zhao et al., 2017) enzymes have both been reported to be associated to the mycoplasma membrane and in close contact with the host cells.

In our hands, close contact between *M. bovis* and the host cells is not required for cytotoxicity, at least for actively dividing cells. Recent evidences support the cytoplasmic localization of GlpO in members of the *Mycoplasma mycoides* cluster (Schumacher et al., 2019), thus raising questions regarding the mechanisms facilitating the release of H_2_O_2_ from mycoplasma cells. Recent studies on different organisms have highlighted the role of water-permeable aquaporins in the transmembrane diffusion of H_2_O_2_ across biological membranes (Bienert and Chaumont, 2014). Interestingly, transposon mutagenesis identified a putative membrane protein of the major facilitator superfamily (MFS) among candidate genes involved H_2_O_2_ production in *M. bovis*. Whether members of the MFS in mycoplasmas may facilitate the export of H_2_O_2_ remains to be demonstrated, but the occurrence of such transporter would be consistent with the absence of H_2_O_2_-degrading enzymes in these organisms.

The contribution H_2_O_2_ production to the virulence of pathogenic mycoplasma species is still controversial (Szczepanek et al., 2014; Schumacher et al., 2019), and the role of eDNA and H_2_O_2_ production in *M. bovis* pathogenesis needs to be confirmed *in vivo*. However, our study provides insight into the mechanism responsible for *M. bovis* host cell cytotoxicity, clearly demonstrating that H_2_O_2_ production is independent from glycerol metabolism but required eDNA. The importance of eDNA in the biology of *M. bovis* is consistent with functional genomic studies with several ruminant mycoplasma species that identified nucleotide metabolism as essential for their survival in cell culture, while dispensable for axenic growth (Baranowski et al., 2010; Skapski et al., 2011; Josi et al., 2019). Finally, nucleases and polynucleotide binding proteins are common component of the mycoplasma surface, besides their role in the acquisition of nutrients these surface proteins are also emerging as potential virulence factors (Minion and Goguen, 1986; Minion et al., 1993; Bendjennat et al., 1999; Jarvill-Taylor et al., 1999; Schmidt et al., 2007; Li et al., 2010, 2018, 2019a; Somarajan et al., 2010; Szczepanek et al., 2010; Browning et al., 2011; Cacciotto et al., 2013, 2019; Masukagami et al., 2013; Sharma et al., 2015; Xu et al., 2015; Yacoub and Mardassi, 2016; Zhang et al., 2016; Gondaira et al., 2017; Yamamoto et al., 2017; Mitiku et al., 2018; Qin et al., 2019).

### *M. bovis* Is Frequently Exposed to eDNA *in vivo*

During its replication in the natural host, *M. bovis* can be frequently exposed to eDNA (Figure 5). This ubiquitous material of terrestrial and aquatic environments can also be isolated from many biological fluids (Vorkapic et al., 2016; Aucamp et al., 2018; Nagler et al., 2018). In normal and diseased tissues, necrosis and apoptosis are the main sources of eDNA, together with autophagy and pyroptosis (Aucamp et al., 2018). Besides these cellular breakdown mechanisms, eDNA can be actively released from living cells either encapsulated in vesicles or in the form of DNA/RNA lipoprotein complexes, termed as virtosomes (Aucamp et al., 2018). Invading pathogens are also contributing to the release of cellular DNA, either directly by damaging the integrity of the infected tissues or indirectly as the outcome of the host immune responses (see below). Ultimately, a significant part of eDNA in the host is also provided by microbial communities (Vorkapic et al., 2016; Nagler et al., 2018).

**FIGUER 5:**
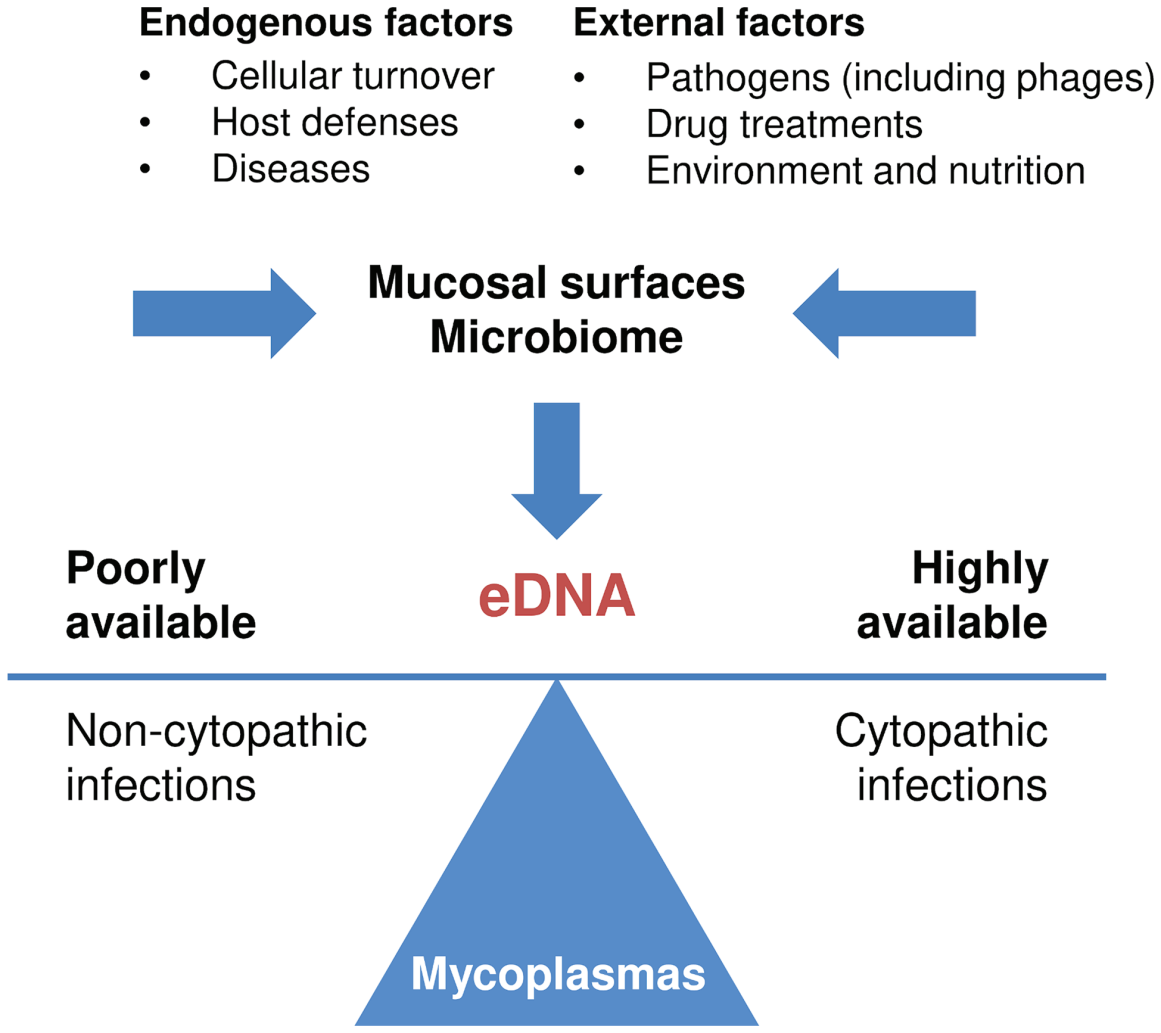
Main sources of eDNA in the host and its influence on *M. bovis* cytotoxicity. The main sources of endogenous and exogenous eDNA are indicated (Aucamp et al., 2018). *M. bovis* is able to induce a pronounced cytopathic effect when a large amount of eDNA is available.

Mycoplasmas have a predilection for mucosal surfaces of the respiratory and genital tracts. The rapid turnover of epithelial cells may thus provide an important source of eDNA to these organisms. *M. bovis* is a re-emerging cause of pneumonia and mastitis in cattle. This economically important species is associated with the bovine respiratory disease complex, a multifactorial disease resulting from complex interactions between environmental, management and host factors, as well as from viral and bacterial co-infections. Viral co-infections have been shown to enhance pulmonary lesions caused by *M. bovis* (Prysliak et al., 2011). Since viral infections and host defenses are responsible for important damages of host tissues, it would be interesting to evaluate the contribution of DNA release on the development of *M. bovis* pneumonia. Another source of eDNA with particular importance for the control of invading pathogens is the activation and release of neutrophil extracellular traps (NET), a complex network of extracellular fibers, primarily composed of DNA and antimicrobial proteins. Recent studies suggested that the DNAse expressed at the surface of *M. bovis* is a key factor for escaping NETs (Zhang et al., 2016; Gondaira et al., 2017; Mitiku et al., 2018). Our data further suggest that *M. bovis* can take advantage of NETs for its proliferation in the animal host, as previously suggested for the swine pathogen *Mycoplasma hyopneumoniae* that was found able to degrade macrophage extracellular traps and incorporate those host nucleotides into its own DNA (Henthorn et al., 2018). The eDNA is also an important structural component of the biofilm matrix raising interesting questions about the interplay between mycoplasmas and microbial communities. Finally, it is noteworthy that bacteriophages and antimicrobial drugs are contributing to the release of massive amounts of prokaryotic DNA.

This study confirms the multifaceted role of eDNA in microbial communities and further identifies this ubiquitous material as a nutritional trigger of *M. bovis* cytotoxicity. *M. bovis* may thus take advantage of the multiple sources of eDNA *in vivo* to modulate its interaction with host cells (Figure 5), a way to overcome its limited coding capacity.

## Data Availability Statement

The datasets generated for this study can be found in the GenBank database under the accession number VSDF00000000 (BioProject: PRJNA556134; BioSample: SAMN12340981).

## Author Contributions

AG, CC, and EB contributed conception and design of the study. XZ, LG, and M-CH were involved in cell culture studies. AB and ES were involved in the construction of the mutant library. ED-F and LN performed sequence analysis. XZ, SA, and RM were involved in mycoplasma cultures. EB wrote the first draft of the manuscript. XZ and ED-F wrote sections of the manuscript. All authors contributed to manuscript revision, read, and approved the submitted version.

### Conflict of Interest

The authors declare that the research was conducted in the absence of any commercial or financial relationships that could be construed as a potential conflict of interest.

## Footnotes

^1^https://www.bioinformatics.babraham.ac.uk/projects/fastqc/

^2^https://blast.ncbi.nlm.nih.gov/Blast.cgi
