# Supplementary material for: Extracellular DNA: A Nutritional Trigger of Mycoplasma bovis Cytotoxicity
Source: Front Microbiol. 2019 Nov 29;10:2753. doi: 10.3389/fmicb.2019.02753 (PMC6895004; doi:10.3389/fmicb.2019.02753)
Supplement: Supplementary file 1 [file Presentation_1.PPTX]

## Slide 1
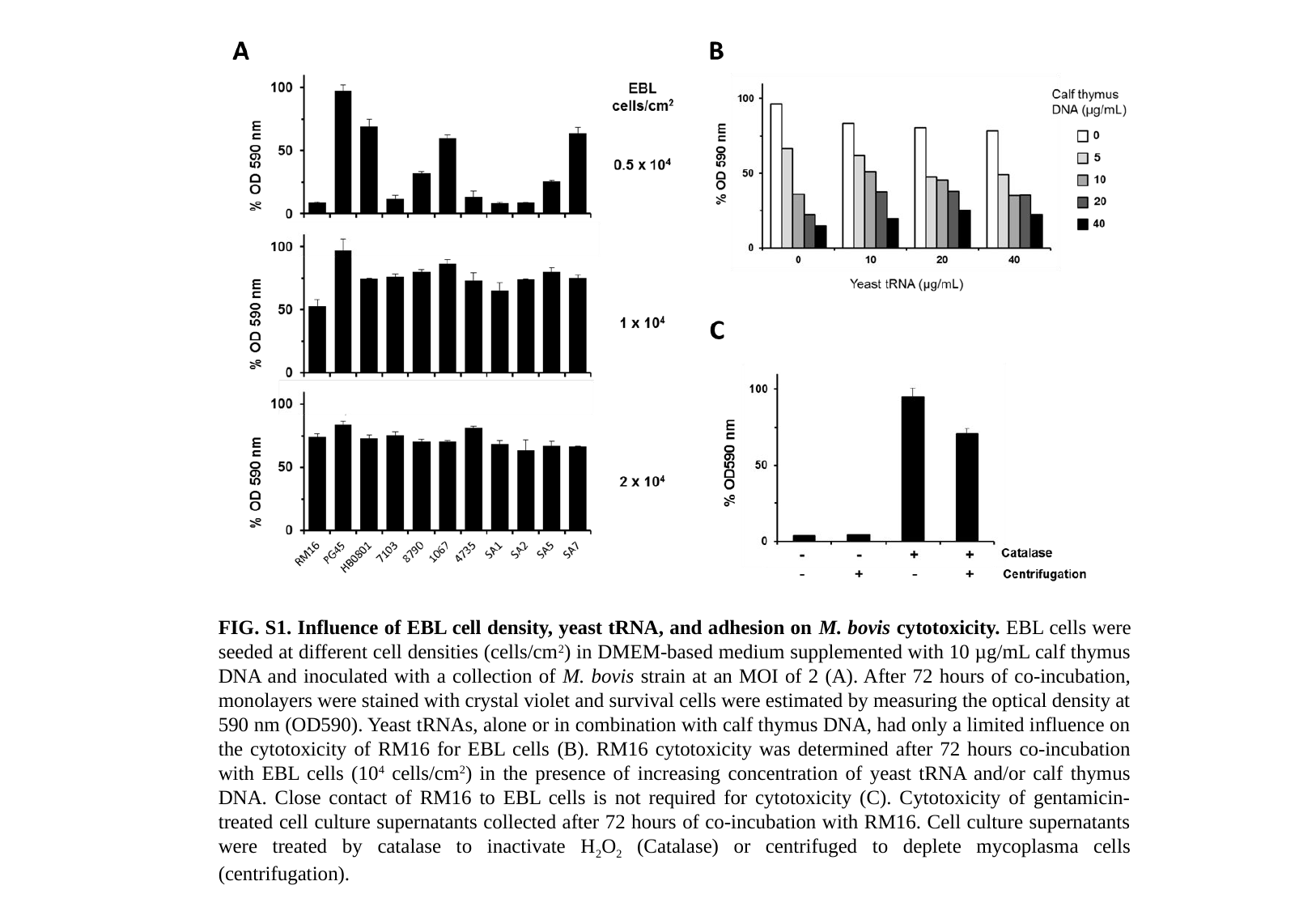

FIG. S1. Influence of EBL cell density, yeast tRNA, and adhesion on M. bovis cytotoxicity. EBL cells were seeded at different cell densities (cells/cm2) in DMEM-based medium supplemented with 10 µg/mL calf thymus DNA and inoculated with a collection of M. bovis strain at an MOI of 2 (A). After 72 hours of co-incubation, monolayers were stained with crystal violet and survival cells were estimated by measuring the optical density at 590 nm (OD590). Yeast tRNAs, alone or in combination with calf thymus DNA, had only a limited influence on the cytotoxicity of RM16 for EBL cells (B). RM16 cytotoxicity was determined after 72 hours co-incubation with EBL cells (104 cells/cm2) in the presence of increasing concentration of yeast tRNA and/or calf thymus DNA. Close contact of RM16 to EBL cells is not required for cytotoxicity (C). Cytotoxicity of gentamicin-treated cell culture supernatants collected after 72 hours of co-incubation with RM16. Cell culture supernatants were treated by catalase to inactivate H2O2 (Catalase) or centrifuged to deplete mycoplasma cells (centrifugation).
