# Supplementary material for: Extracellular DNA: A Nutritional Trigger of Mycoplasma bovis Cytotoxicity
Source: Front Microbiol. 2019 Nov 29;10:2753. doi: 10.3389/fmicb.2019.02753 (PMC6895004; doi:10.3389/fmicb.2019.02753)
Supplement: Supplementary file 2 [file Presentation_2.PPTX]

## Slide 1
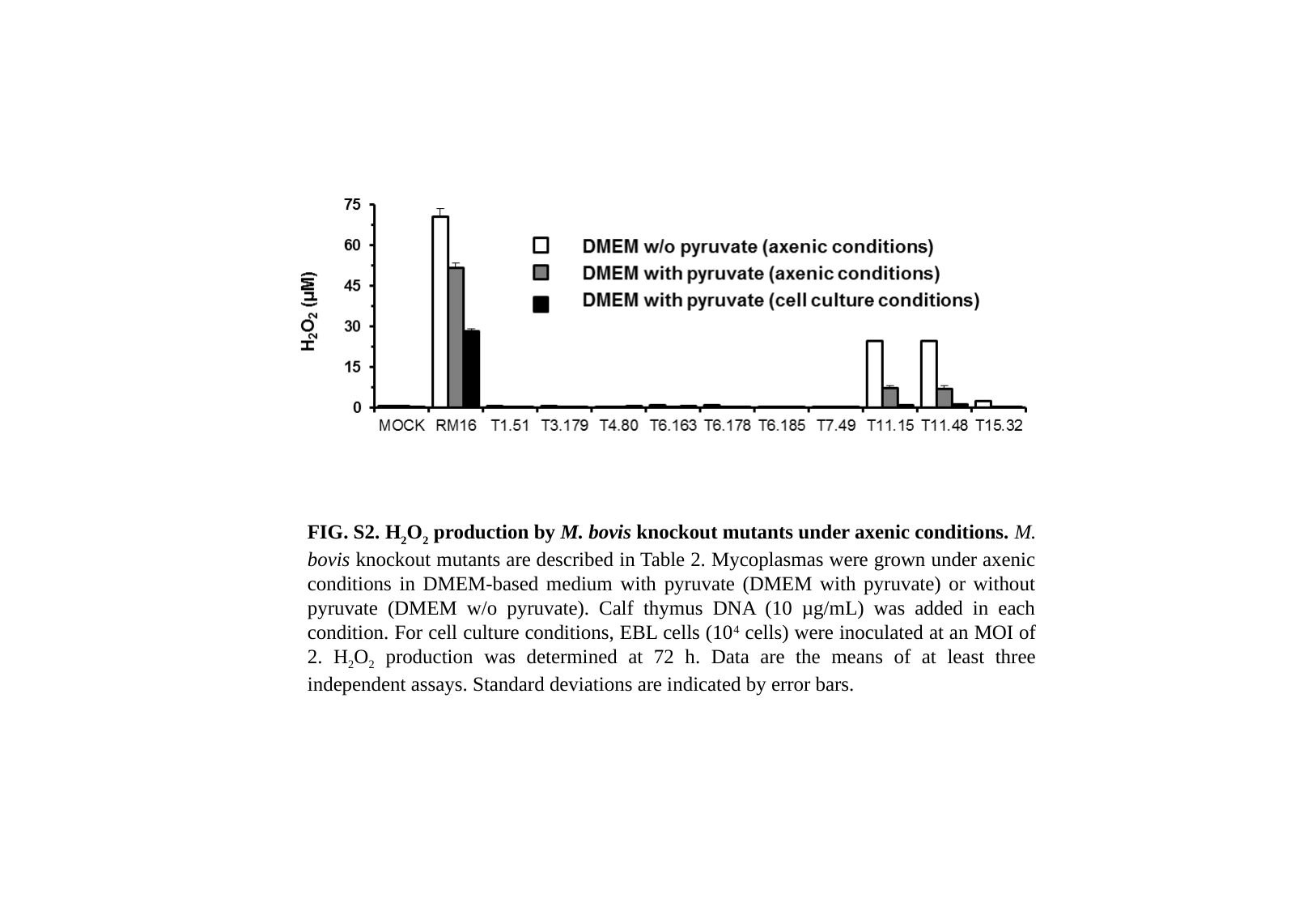

FIG. S2. H2O2 production by M. bovis knockout mutants under axenic conditions. M. bovis knockout mutants are described in Table 2. Mycoplasmas were grown under axenic conditions in DMEM-based medium with pyruvate (DMEM with pyruvate) or without pyruvate (DMEM w/o pyruvate). Calf thymus DNA (10 µg/mL) was added in each condition. For cell culture conditions, EBL cells (104 cells) were inoculated at an MOI of 2. H2O2 production was determined at 72 h. Data are the means of at least three independent assays. Standard deviations are indicated by error bars.
